# Enhancing Stability of Microwave‐Synthesized Cs_2_Sn_x_Ti_1‐x_Br_6_ Perovskite by Cation Mixing

**DOI:** 10.1002/cssc.202402073

**Published:** 2024-12-19

**Authors:** Emmanuel Reyes‐Francis, Beatriz Julián‐López, Carlos Echeverría‐Arrondo, Jhonatan Rodríguez‐Pereira, Diego Esparza, Tzarara López‐Luke, Jaime Espino‐Valencia, Daniel Prochowicz, Iván Mora‐Seró, Silver‐Hamill Turren‐Cruz

**Affiliations:** ^1^ Instituto de Investigación en Metalurgia y Materiales Universidad Michoacana de San Nicolás de Hidalgo Edificio U, Ciudad Universitaria Morelia, Mich C.P. 58030 México; ^2^ Institute of Advanced Materials (INAM) Universitat Jaume I Av. Sos Baynat, s/n 12071 Castelló de la Plana España; ^3^ Center of Materials and Nanotechnologies Faculty of Chemical Technology University of Pardubice Nam. Cs. Legii 565 Pardubice 53002 Czech Republic; ^4^ Central European Institute of Technology Brno University of Technology Purkyňova 123 Brno 612 00 Czech Republic; ^5^ Unidad Académica de Ingeniería Eléctrica Universidad Autónoma de Zacatecas Jardín Juárez 147, Zacatecas Centro Zacatecas, Zacatecas C.P. 98000 México; ^6^ Facultad de Ingeniería Química División de Estudios de Posgrado Universidad Michoacana de San Nicolás de Hidalgo, Edificio Q, Edificio U, Ciudad Universitaria Morelia, Mich C.P. 58030 México; ^7^ Institute of Physical Chemistry Polish Academy of Sciences Kasprzaka 44/52 01–224 Warsaw Poland

**Keywords:** Cesium titanium tin bromide perovskite, Microwave irradiation synthesis, Lead-free double perovskite, Outdoor stability

## Abstract

The double‐perovskite material Cs_2_TiBr_6_ shows excellent photovoltaic potential, making it a promising alternative to lead‐based materials. However, its high susceptibility to degradation in air has raised concerns about its practical application. This study introduces an interesting synthesis approach that significantly enhances the stability of Cs_2_TiBr_6_ powder. We implemented a gradual cation exchange process by substituting Ti^4+^ with Sn^4+^ in the efficient microwave‐assisted synthesis method, developing a double perovskite Cs_2_Sn_x_Ti_1‐x_Br_6_ type. A systematic study of increasing concentration of Sn^4+^ in Cs_2_TiBr_6_ perovskite has been performed to analyze the effect of Sn‐doping degree on the chemical and thermal stability of the material and the optical features in both nitrogen and ambient atmospheres, significantly increasing the stability of the material in the air for over a week. Furthermore, introducing Sn^4+^ results in a more uniform polygonal crystal morphology of the powders and a slight band gap broadening. We show that microwave‐assisted synthesis is highly efficient and cost‐effective in producing more sustainable lead‐free perovskite materials with enhanced stability and desirable electrical characteristics. This work suggests a promising method for synthesizing perovskite materials, opening new routes for scientific research and applications.

## Graphical abstract

This research introduces a microwave radiation synthesis technique for synthesizing Cs_2_Sn_x_Ti_1‐x_Br_6_ lead‐free double perovskite powders. By incorporating Sn^4+^ cations, the stability of the material is improved, particularly at elevated temperatures and outside conditions. This efficient and economical synthesis method holds great potential to significantly enhance the properties of environmentally friendly perovskite materials, promoting advancements in perovskite technology.

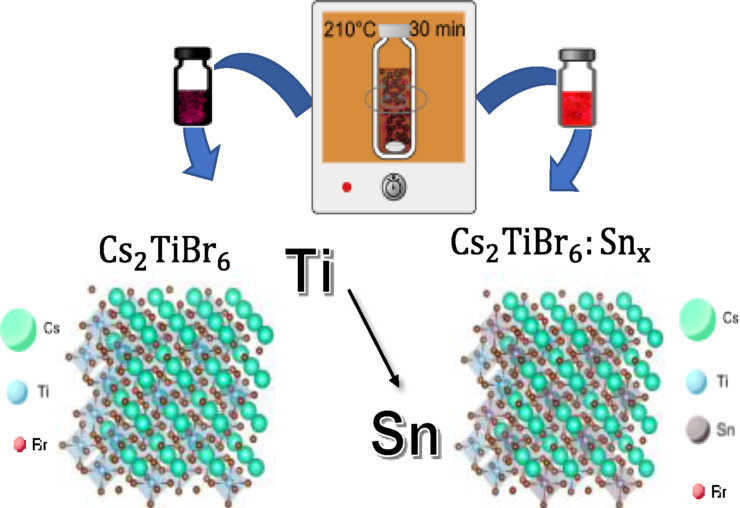

## Introduction

The progress of Sn‐based perovskite solar cells (PSCs) in just a few years has been remarkable, with single‐junction solar cells having attained power conversion efficiency (PCE) values of over 26 %.^[1]^ However, regulatory concerns regarding lead toxicity and the instability of these perovskite materials under ambient conditions present significant challenges to their commercialization.^[8]^

These accomplishments highlight the steadfast commitment of the scientific community toward exploring alternative Pb‐free perovskite materials to mitigate toxicity concerns while keeping high device efficiencies.^[11]^ Indeed, single and double vacancy‐ordered (A_2_BX_6_) structures incorporating environmentally more benign B cations, such as Sn^2+^,[^1^, ^16^] Sb^3+^,^[21]^ Bi^3+^,^[25]^ Ti^4+^,^[31]^ or Ag^+^ is at the forefront of lead‐free perovskite research for photovoltaic and optoelectronic applications. These materials exhibit similar structural and chemical properties to traditional halide perovskites but avoid containing lead.[^31^, ^41^] Among lead‐replacement candidates, titanium‐based vacancy‐ordered double perovskite has attracted particular attention due to its elemental titanium abundance and low toxicity, a non‐critic element, which makes it a promising alternative” Consequently, several research groups have successfully synthesized Cs_2_TiBr_6_ utilizing different techniques.^[21]^

Our prior research entailed synthesizing pristine Cs_2_TiBr_6_ perovskite powder using a straightforward, low‐energy, scalable microwave (MW) – mediated route.^[24]^ This approach relies on uniform heating, accelerating reaction rates, ensuring a high phase purity, and accelerating the product formation over a condensed timeframe. The MW method effectively produces high‐quality perovskite materials with lower defects and minimal impurities, overcoming some limitations (high‐vacuum, high temperature, high‐energy milling, multistep solution processes, etc.) associated with the previously reported syntheses, constraints that hinder industrial scalability.^[25]^

However, when Cs_2_TiBr_6_ perovskite is exposed to air at different atmospheric conditions and times, it undergoes decomposition, leading to CsBr and amorphous TiO_2_.^[24]^ Avoiding this degradation would mean a critical advancement in the accessibility of efficient and stable lead‐free perovskite materials. Recent investigations by Konstantatos and co‐workers demonstrated the improvement in the stability of Cs_2_TiBr_6_ nanocrystals through a SnBr_4_ surface treatment and by making mixed Cs_2_Ti_1‐x_Sn_x_Br_6_ compositions.^[29]^ They found that the compound stabilized at high Sn percentages (x~60 %). These works employed a colloidal route through long vacuum/nitrogen cycles and hot injection. The literature on this subject indicates that understanding the degradation process and its relationship to the synthesis methodology is crucial for advancing the development of the material′s stability in this system.[^13^, ^15^, ^31^]

The potential for mixing tin (Sn) cations with titanium (Ti) arises from the intrinsic properties of tin ions. For instance, tin commonly exhibits ^2+^ and ^4+^ oxidation states, allowing it to achieve a noble gas configuration, whereas titanium is mainly stabilized in the ^4+^ oxidation state.^[35]^ Additionally, the larger ionic radius of tin ions effectively shields the nuclear charge. In contrast, titanium ions, which are smaller in higher oxidation states, experience a stronger electrostatic attraction, potentially compromising their stability.^[37]^

In this work, we demonstrate that introducing Sn^4+^ through an MW‐accessible synthesis can significantly enhance the stability of Cs_2_TiBr_6_ powder. The result is a solid solution of Cs_2_Ti_x_Sn_1‐x_Br_6_ with varying proportions of Sn^4+^ (where x ranges from 0 to 0.75). We utilized structural and optoelectronic techniques to evaluate atmospheric stability levels. The results showed that ambient stability increased from 12 hours for pristine Cs_2_TiBr_6_ (x=0) to one week for the X=0.75 Sn−Ti perovskite composition.

## Results and Discussion

**Figure 1:**
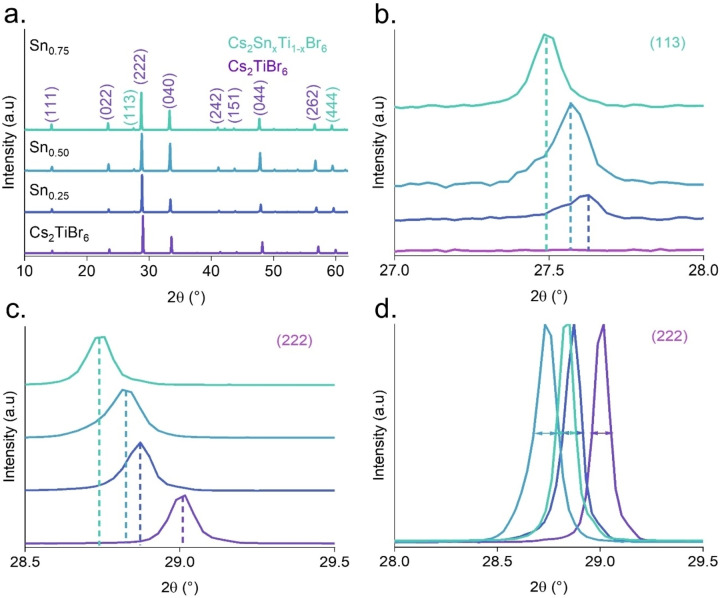
Cs_2_TiBr_6_ and Cs_2_Sn_x_Ti_1‐x_Br_6_ powders over the MW synthesis. a) Evolution of XRD patterns. b) XRD analysis of the Ti (222) peak. c) XRD analysis of the Ti (222) peak, and d) Magnification of the indexed peak (222) of Cs_2_TiBr_6_ Illustrating the change in FWHM.

Mixed Cs_2_Ti_x_Sn_1‐x_Br_6_ (x=0, 0.25, 0.5, and 0.75) perovskite powders were prepared according to the microwave‐mediated (MW) synthesis schematically depicted in Figure S1. Figure 1a shows the X‐ray diffraction patterns of the pristine Cs_2_TiBr_6_ and the mixed Cs_2_Sn_x_Ti_1‐x_Br_6_ powders (further denoted as Sn_0.25_, Sn_0.50_, and Sn_0.75_) with increasing amounts of Sn^4+^. The Cs_2_TiBr_6_ sample exhibits diffraction peaks at 14.51, 23.53, 28.96, 33.59, 40.89, 41.45, 47.73, 49.75, 53.74, and 57.18°, corresponding to the (111), (022), (222), (040), (242), (151), (044), (153), (062) and (262) crystallographic planes of the *Fm*3*m* space group symmetry, as expected for vacancy ordered double perovskites such as Cs_2_TiBr_6_. Meanwhile, the mixed‐cation samples exhibit two new peaks at 27.3 and 59.7°, corresponding to the (113) and (444) crystallographic planes. These peaks are intensified in the samples with higher amounts of Sn^4+^ cations.^[30]^ The indexed peaks show a trend towards lower 2θ values as the quantity of Sn^4+^ increases, as shown in Figure 1b. Upon examining Figure 1c, the main peak of Cs_2_TiBr_6_ (222) experiences a decrease in 2θ angle, being 29.02°, 28.87°, 28.83°, and 28.74° for the original Cs_2_TiBr_6_ sample and the solid solutions with Sn_0.25_, Sn_0.50_, and Sn_0.75_, respectively. This behavior confirms that the crystal lattice expands upon adding Sn^4+^ cations. This expansion occurs because the ionic radius of the Sn^4+^ (81 pm) is larger than the Ti^4+^ (74.5 pm) in octahedral coordination. The lattice parameters were calculated using Bragg′s law, resulting in values of 10.65 Å, 10.72 Å, 10.73 Å, and 10.74 Å, respectively. As expected, the crystal size increased proportionally with the addition of Sn^4+^ due to the variation in the ionic radii of the cations. Also, the lattice constants of mixed perovskites, composed of two perovskite crystals with similar lattice constants, are expected to comply with Vegard′s law and exhibit a linear relationship with their compositional order. The lattice parameter increases linearly with the Sn content, as represented in Figure S2, in good agreement with the empirical Vegard′s Law. These values suggest that Sn^4+^ is incorporated into the perovskite structure during synthesis Figure 1d illustrates the full width at half maximum (FWHM) of the (222) peak. The FWHM values for the pristine Cs_2_TiBr_6_ and solid solutions of Sn_0.25_, Sn_0.50,_ and Sn_0.75_ samples are 0.0922, 0.0998, 0.1271, and 0.1015°, respectively. As the concentration rises, the widening of FWHM values indicates a growth in particle size. We analyzed these results using simulated calculations. The simulated and experimental XRD diffractograms match precisely, indicating congruence between the experimental results and simulations, see Figure S3. In this case, when simulating the addition of more Sn^4+^ than Ti^4+^ ions in this scenario, the two peaks (113) and (444) in Sn‐rich compounds become evident, and their intensity increases, aligning with the experimental XRD results.

On the other hand, the perovskite powder deposited in the vial changes coloration from a dark red color to a lighter reddish pigmentation with the gradual increment of Sn^4+^. The crystalline structure of the mixed‐cation perovskites is shown in Figure S3. These findings are crucial for understanding the behavior of mixed cations over the crystalline structure.

**Figure 2:**
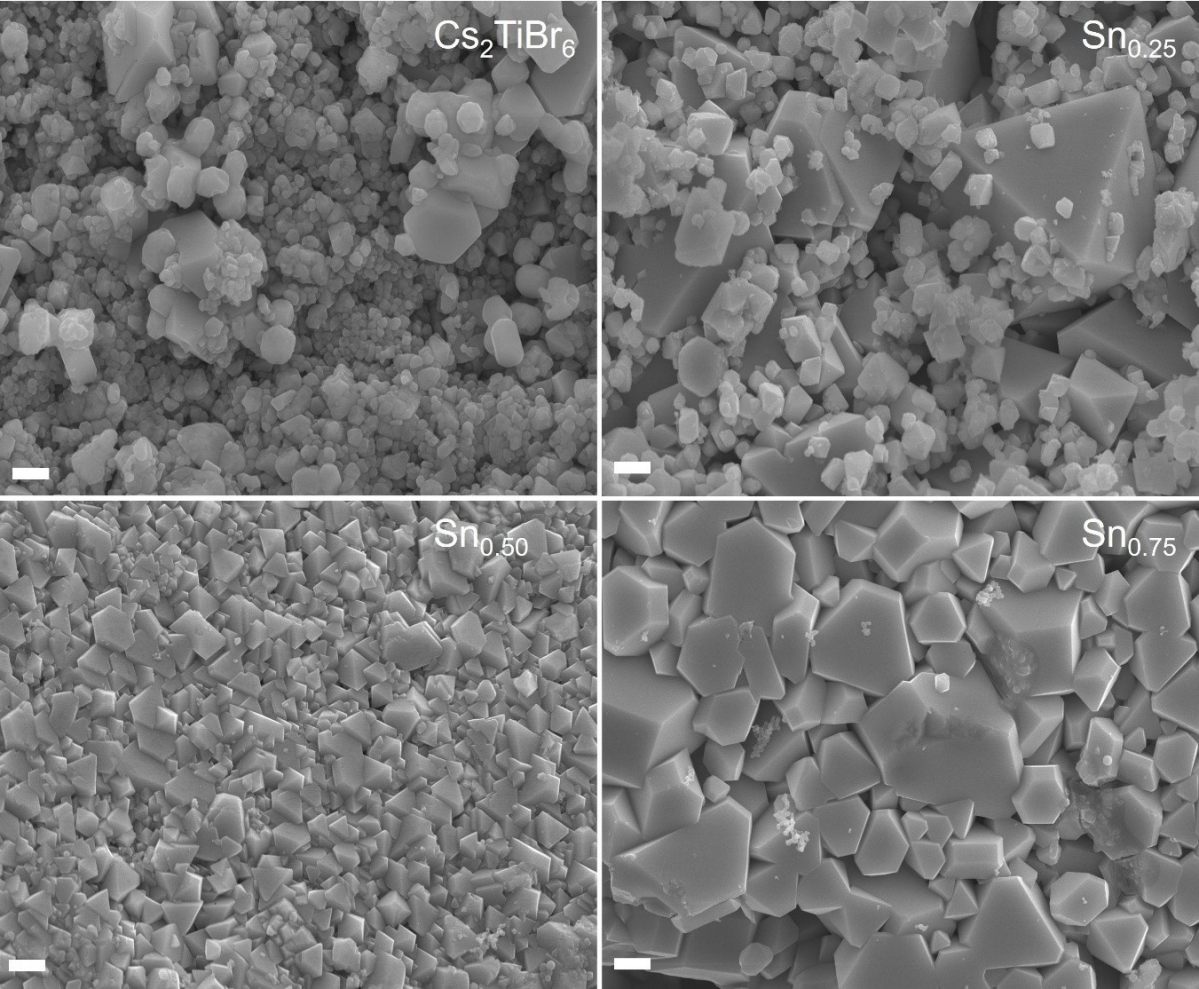
SEM micrographs of the pristine and mixed Cs_2_Sn_x_Ti_1‐x_Br_6_ double perovskite samples. The scale bar represents 1 μm.

To gain an understanding of how the introduction of Sn^4+^ modifies the mixed Cs_2_Sn_x_Ti_1‐x_Br_6_ system, we analyzed the morphological alterations by scanning electron microscopy (SEM). We found that the incorporation of Sn^4+^ significantly impacts the morphology of the resulting material. Figure 2 showcases that the increasing amount of Sn^4+^ in the Cs_2_TiBr_6_ resulted in larger 1 to 3 mm polyhedral crystals with higher definition and uniformity. This contrasts with the spherical agglomerates of hundreds of nanometers in size, dominating the pure Cs_2_TiBr_6_ sample. The addition of Sn^4+^ led to a transition towards well‐defined polyhedral forms and an increase in the quality of the crystal shapes, concomitantly with a reduction of spherical Cs_2_TiBr_6_ agglomerates. The compositional analysis was also performed using energy‐dispersive X‐ray spectroscopy (EDS). The EDS spectra, shown in Figure S4, illustrate the relative atomic proportions of Cs, Ti, Sn, and Br for the different samples. A perfect match was found between the experimental composition and that expected from the stoichiometric ratio of Cs_2_Ti_1‐x_Sn_x_Br_6_ mixed‐cation perovskites.

To obtain information on the photophysical properties, we measured the absorption spectra of the pristine Cs_2_TiBr_6_ and mixed‐cation Cs_2_Sn_x_Ti_1‐x_Br_6_ powders (Figure S5a). The analysis reveals that incorporating Sn^4+^ in concentrations over Sn_0.25_ elemental proportion is correlated with a slight deviation towards a longer wavelength, around 700 nm. Adding Sn_0.25_ to the sample is almost identical to the pristine sample. However, there is a slight absorption increase around the 850 nm region. This prominence expands and adapts in response to adding a higher quantity of Sn^4+^ ions. In addition, a slight change in the absorption pattern was observed in the Sn_0.50_ sample. This change becomes evident when the sample has a Sn_0.75,_ resulting in a slight shift in the spectrum. It is speculated that the shallow absorption zone is associated with a phonon‐assisted mechanism.^[41]^ Regardless, except for what was mentioned, the optoelectronic properties of the pristine and hybrid perovskite materials remain the same. However, as shown in Figure S5b, The bandgap values of the pristine and mixed‐cation Cs₂Sn_x_Ti₁_‐x_Br₆ samples were determined using the Tauc plot fitting method. The calculated bandgap values were 1.81 eV for the pristine sample, 1.81 eV for Sn₀.₂₅, 1.82 eV for Sn₀.₅₀, and 1.83 eV for Sn₀.₇₅. These results indicate that the variation in optical properties among the samples is negligible.

**Figure 3:**
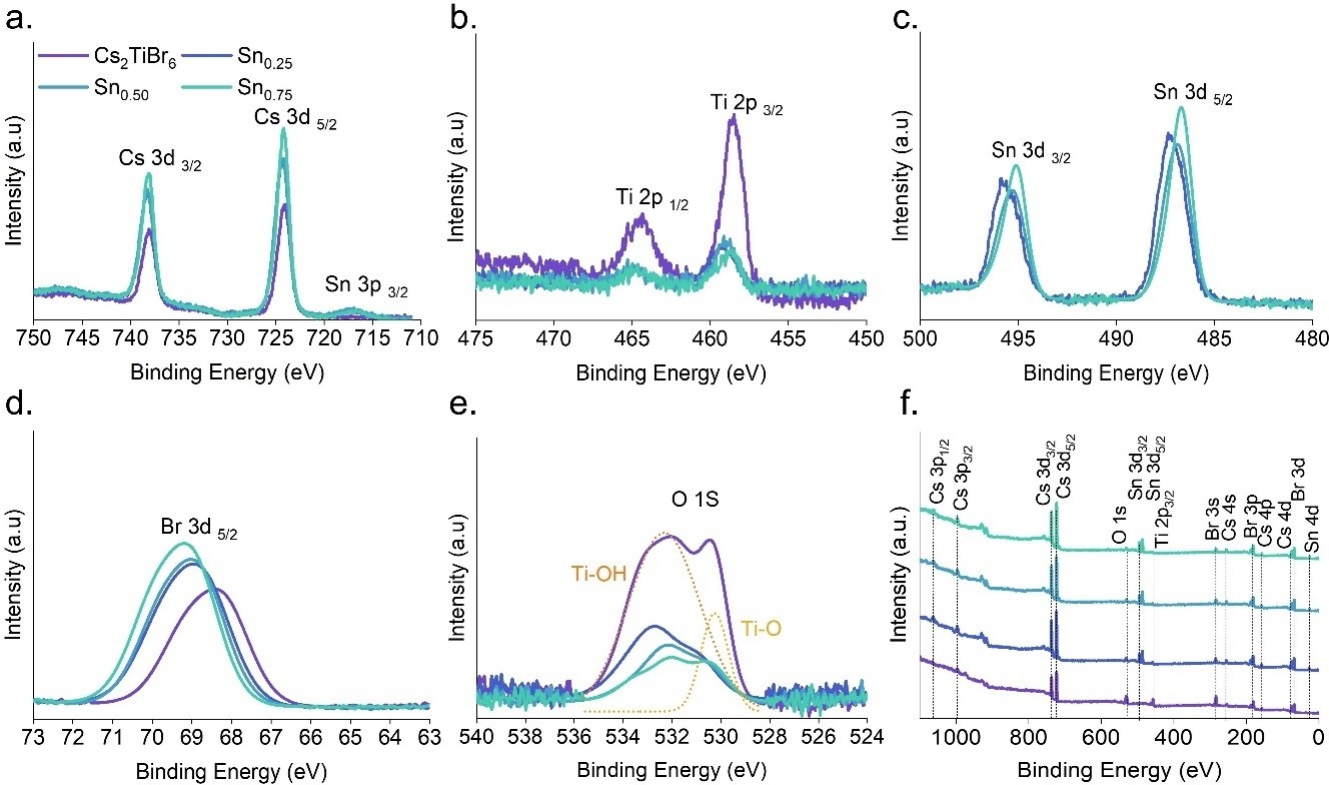
XPS spectra of the hybrid Cs_2_Sn_x_Ti_1‐x_Br_6_ powders synthesized by MW: a) Cs *3d*, b) Ti *2p*, c) Sn *3d*, d) Br *3d*, and e) O 1s (adding deconvolution of the sample′s various oxygen species f) X‐ray photoelectron spectroscopy (XPS) survey spectra.

To evaluate the powdered material′s bonding properties and chemical composition states, we used X‐ray photoelectron spectroscopy (XPS) measurements. In Figure 3a, the Cs 3d spectrum displays two distinct peaks at 737.9 and 724.2 eV energy levels, corresponding to the Cs 3d_3/2_ and Cs 3d_5/2_ orbitals. Adding Sn leads to a slight increase in binding energy. Furthermore, in Figure 3b, we find two distinct peaks at 458.61 and 464.7 eV ascribed to the Ti 2p_1/2_ and Ti 2p_3/2_ orbitals, respectively. Including Sn^4+^ leads to a progressive reduction in the intensity of the Ti 2p_3/2_ and Ti 2p_1/2_ orbitals. Conversely, in Figure 3c, the Sn_x_ samples exhibit two distinct peaks within the 495–496 and 487–488 eV ranges, corresponding to the Sn 3d_3/2_ and Sn 3d_5/2_ orbitals, respectively. This evidences the presence of Sn species and that when the quantity of Sn ions increases, the orbitals experience a binding energy shift, contrary to the behavior observed in Ti. These results are consistent with the EDS observations confirming that Sn cations substitute Ti ones in the crystal lattice in the Cs_2_Sn_x_Ti_1‐x_Br_6_ structure. In addition, the Br 3d spectra of the powders depicted in Figure 3d display a prominent peak at 68.38 eV, corresponding to the orbital Br 3d_5/2_. Moreover, there is a notable transition towards a higher binding energy as the concentration of Sn^4+^ increases. Since chemical shift values depend on the degree of electron bond polarization between nearest‐neighbor atoms, these results evidence significant variations in the nature of M−Br bonds (M: Ti, Sn). Considering the electronegativities of the elements (2.96 for bromine, 1.32 for titanium, and 1.89 for tin), the higher the difference in electronegativity (cation‐anion), the higher the ionicity of the bond. Thus, the more polarized Ti−Br bonds induce a higher separation of charges (positive charge density on the cation and negative charge density on the anion) than in the case of Sn−Br bonds. This has two main consequences: (i) Ti is more acid cation than Sn according to Lewis acid‐base theory, and (ii) the Br^−^ anion is more acid (i. e. more positive or less negative) in the mixed Cs_2_Sn_x_Ti_1‐x_Br_6_ perovskite, which explains the higher binding energy of Br 3d_5/2_ peak as Sn content increases.

The O 1s spectra, shown in Figure 3e, possess distinct peaks at 532.4 and 530.16 eV. The signal found at 532.4 eV is mainly caused by the presence of Ti‐OH‐type bonds and organic O, which result from the adsorption of water molecules on the material′s surface and adventitious carbon. In the Sn_x_ samples, the intensity of this degradation gradually diminishes. Conversely, the signal observed at 530.2 eV is ascribed to a Ti−O bond, suggesting the presence of titanium oxide caused by the air′s moisture. This also diminished progressively in Sn_x_ materials. These results suggest that adding Sn^4+^ to the mixed Sn−Ti perovskite protects against moisture, preventing the anchoring of water at the material′s surface and ambient oxidation, with the subsequent perovskite decomposition. Figure 3f shows the XPS spectra, confirming the presence of Cs, Ti, and Br in all samples and Sn in the crystalline structure of mixed perovskites. It is observed that when there is an increase in Sn^4+^ ions, the amount of Ti^4+^ decreases. The increase in Sn^4+^ concentration resulted in a corresponding decrease in the quantity of Ti, confirming the Cs_2_Sn_x_Ti_1‐x_Br_6_ type structure.

**Figure 4:**
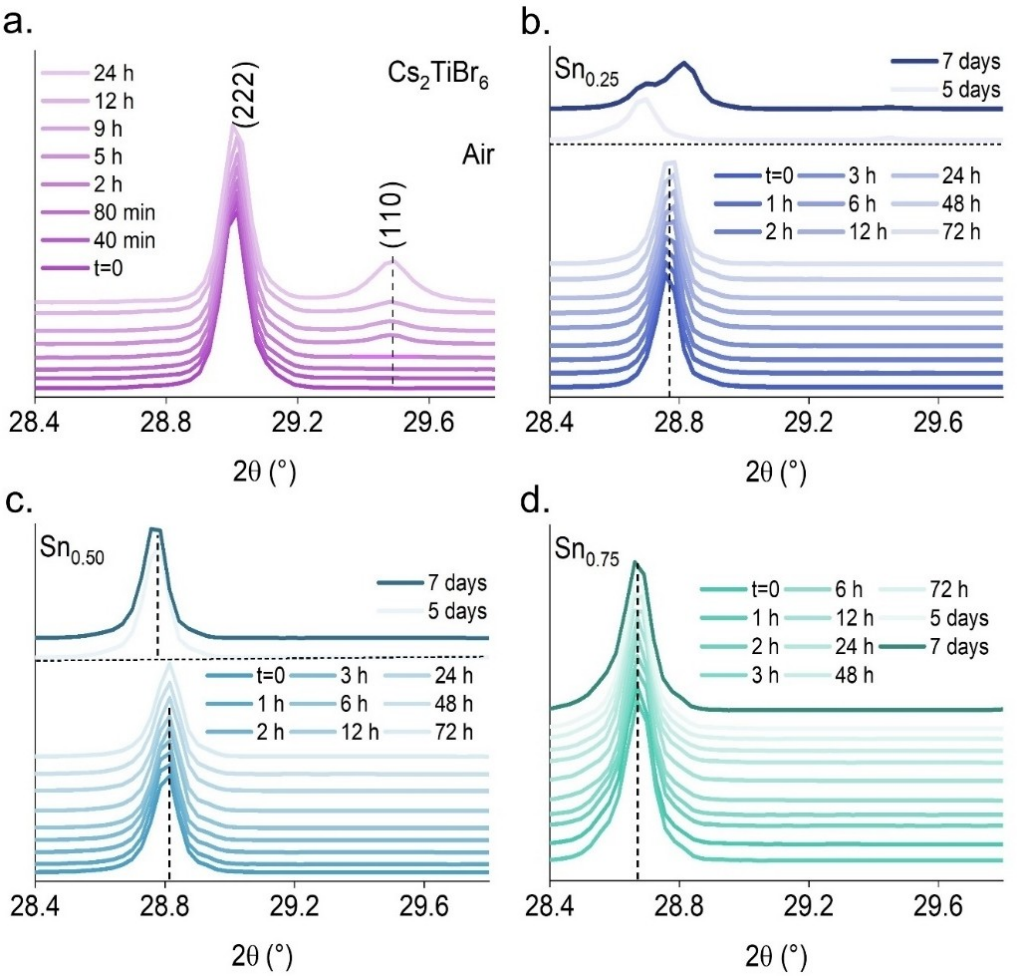
Evolution of the XRD patterns of the main reflection Cs_2_TiBr_6_ (222) for pristine and Cs2Sn_x_Ti_1‐x_Br_6_ powders synthesized by MW over time in an ambient atmosphere (20–23 °C, 30–35 % RH). a) Pristine, b) Sn_0.25_, c) Sn_0.50_ and d) Sn_0.75_.

Considering these observations, we evaluated the stability of these materials to ambient factors. The experiment was conducted under ambient air conditions with a relative humidity of 30–35 %, a temperature range of 20–23 °C, and a different Sn^4+^‐doped mol % concentration. The behavior of the main peak (222) under ambient conditions is examined in Figure 4 before and after exposure to air. The XRD spectra reveal the effect of Sn^4+^ doping on Cs_2_TiBr_6_ stability at different concentrations. According to the results, the samples mixed with Sn^4+^ do not show any significant structural change until a complete week, a much longer time than the pristine sample, see Figure 4a, which shows material degradation after only 5 h. This phenomenon is evident from the increased presence of the CsBr (110) peak, which correlates with the degradation of the perovskite structure, as detailed in our previous study.^[24]^ When the elemental proportion of Sn_0.25_, the material is stable for 3 days, see bottom of Figure 4b. However, the stability level decreases significantly after three days. See the top of Figure 4b, which is indicated by a leftward displacement of the diffraction peak, suggesting the potential degradation of the material into amorphous compounds. Similar stability is observed in Figure 4c for Sn_0.50_. Finally, in Figure 4d, stability remains intact for seven days for Sn_0.75_ concentration. The Sn^4+^ incorporation enhances the stability of the mixed perovskite under ambient conditions. The whole diffraction pattern (10–60°, 2θ) is provided in Figure S6.

The stability of the mixed Sn/Ti perovskites can be attributed to the increasing covalency of the crystalline structure when titanium is replaced by tin cations. As already mentioned in the XPS discussion, the bond charge polarization in Ti−Br is higher than in Sn−Br, and the acidity of Ti(IV) cation is higher than Sn(IV). In addition, the ionic radii of Ti(IV) are smaller than that of Sn(IV), thus concentrating the positive charge density, which also contributes to increasing the Ti acidity. Both effects make the pristine perovskite more prone to be attacked by nucleophilic species such as water or oxygen from air, moisture, etc. Therefore, the increase of Sn in the system improves the chemical stability.

**Figure 5:**
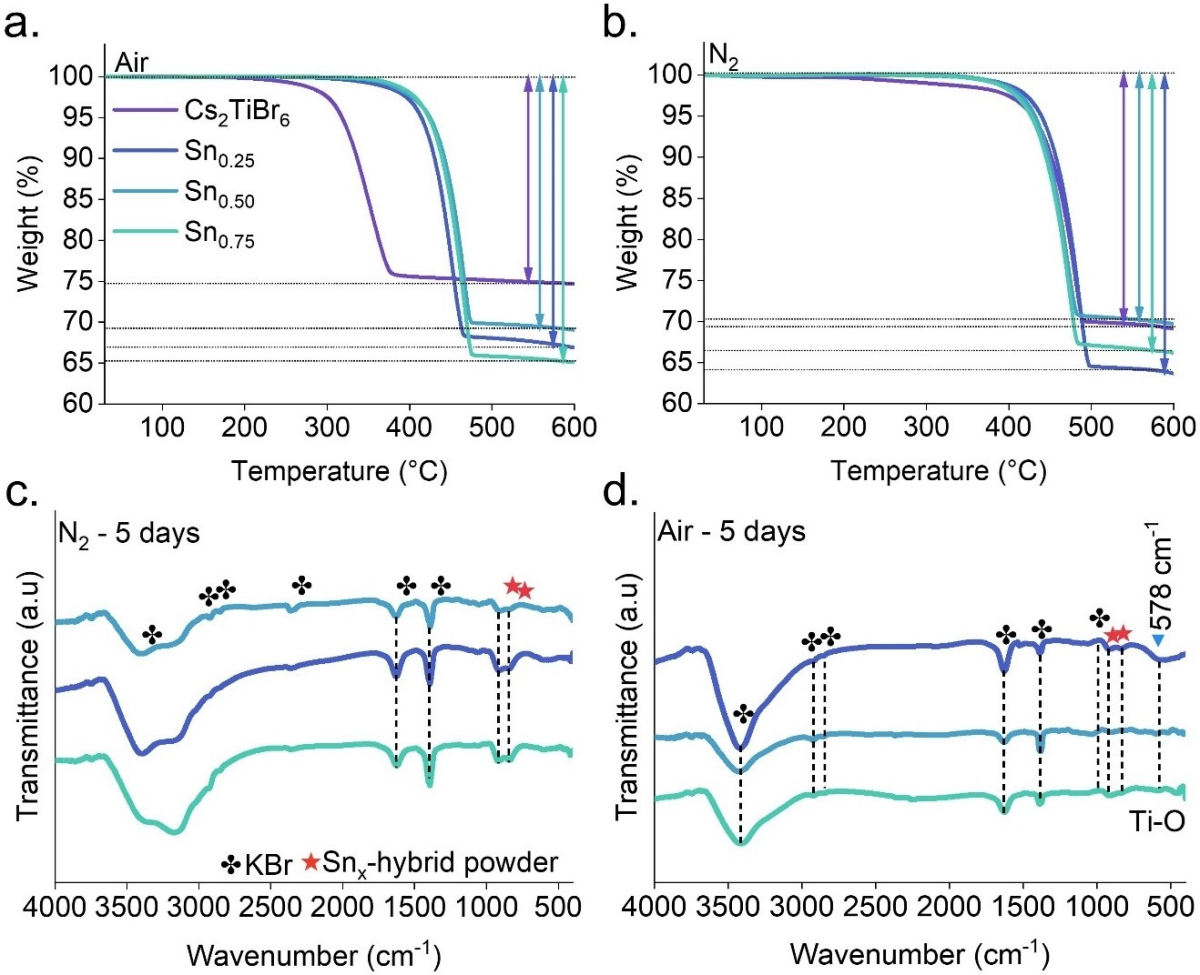
TGA and FTIR spectra of pristine Cs_2_TiBr_6_ and mol % Sn‐doped powders synthesized by MW under a) air and b) nitrogen flows, c) after five days of exposure to nitrogen conditions, and d) after five days of atmospheric conditions.

We conducted thermal gravimetric analysis (TGA) studies to examine the thermodynamic stability of pristine Cs_2_TiBr_6_ and Cs_2_Sn_x_Ti_1‐x_Br_6_ powders. TGA experiments were carried out using nitrogen and air flows to investigate the inherent thermal stability of powders and establish evidence for the reaction of Cs_2_TiBr_6_ and Cs_2_Sn_x_Ti_1‐x_Br_6_ with oxygen, resulting in the formation of residual compounds degraded from Ti and Sn ions. At the initial phase, in Figure 5a, there is a mass loss of around 3 % in all pristine and mixed cation samples in an oxygen atmosphere, up to a temperature of roughly 130 °C. This mass loss is attributed to the evaporation of water that is adsorbed on the surface due to moisture. The degradation of the pristine Cs_2_TiBr_6_ sample starts at around 250 °C. Nevertheless, the mixed‐cation Cs_2_Sn_x_Ti_1‐x_Br_6_ samples exhibit higher thermal stability, delaying the mass loss to temperatures above 400 °C. This outcome indicates that the presence of Sn^4+^ serves as a protective mechanism against the thermal degradation of perovskite powder, drastically delaying its transformation into CsBr and amorphous TiO_2_. It is observed that the stability of the samples against deterioration increases proportionally with the quantity of Sn present. The Cs_2_TiBr_6_ sample exhibits a lower mass loss than Cs_2_Sn_x_Ti_1‐x_Br_6_ in this temperature range under airflow. The fact suggests that the pristine sample has experienced significant oxidative degradation. Conversely, including Sn^4+^ ions in the samples resulted in a higher temperature requirement for the onset of progressive oxidative decomposition. The findings of the TGA analysis conducted in a nitrogen atmosphere are depicted in Figure 5b. Likely to TGA analysis in air, a minor reduction in mass attributed to the presence of absorbed water occurs. Under nitrogen conditions, all samples demonstrated similar thermal stability up to 400 °C. Above this temperature, a significant mass loss was observed. These experiments indicate that oxygen species initiate the degradation mechanism of the pristine perovskite when exposed to air. Notably, the Ti/Sn mixed perovskite degradation occurs at similar temperatures across different atmospheres, suggesting that the presence of Sn^4+^ effectively inhibits the reaction with oxygen species. However, oxidative degradation inevitably occurs above 400 °C. In Table 1 is observed the residual weight of the powder when exposed to air and nitrogen flows for both the Pristine and Sn‐mixed Cs_2_TiBr_6_ samples. These thermal stability results are highly interesting as they surpass temperatures over 300 °C, a critical barrier at which conventional Pb‐based perovskite materials are degraded.

**Table 1:** The residual weight of the powder was measured upon exposure to air and N_2_ flows for both the Pristine and Sn‐doped Cs_2_TiBr_6_ samples.

Sample	Weight loss (%)	
	O_2_	N_2_
Cs_2_TiBr_6_ Pristine	25.23	30.65
Cs_2_Sn_0.25_Ti_0.75_Br_6_	30.76	35.77
Cs_2_Sn_0.50_Ti_0.50_Br_6_	33.08	29.71
Cs_2_Sn_0.75_Ti_0.25_Br_6_	34.76	33.62

Finally, we analyzed Fourier‐transform infrared (FTIR) spectra of Cs_2_Sn_x_Ti_1‐x_Br_6_ samples before and after environmental conditions to demonstrate the stability enhancement in these systems. Figure S7 shows the initial state of the materials, which were mixed with a KBr pellet as a blank, as described in SI. Two characteristic peaks were detected at 927 and 893 cm^−1^, related to the Sn/Ti mixed samples. Figure 5c displays the FTIR spectra of the materials after five days of exposure to an N_2_ atmosphere, which shows that powders can be kept stable in a controlled atmosphere with no changes indicating material breakdown. However, Figure 5d displays the FTIR spectra of the samples after five days of exposure to air, which shows a peak at 578 cm^−1^. This peak corresponds to the Ti−O group′s bending vibration. These results are in good agreement with those shown in Figures S3 and S7.

## Conclusions

We have demonstrated the successful synthesis of mixed‐cation Cs_2_Sn_x_Ti_1‐x_Br_6_ perovskites using a simple microwave‐assisted methodology by varying the Sn content. The efficient incorporation of Sn was probed by XRD and XPS analysis. The amount of tin had an impact on the lattice parameters and the morphology of the sample, but the variation in the optical properties was negligible. The stability of the resulting materials under atmospheric conditions is greatly enhanced, with the sample Sn_0.75_ remaining stable for up to one week in the ambient atmosphere. The less acidic tin cation and the higher covalency of the Sn−Br bonds in the mixed Ti/Sn hybrid perovskites are responsible for the enhancement in the chemical and thermal stability of the system. In conclusion, this synthetic route offers new possibilities for producing alternative Pb‐free perovskite materials. In this study, we revealed that the introduction of Sn^4+^ prevents the degradation of the Ti‐containing perovskite material. This advancement opens new possibilities for developing eco‐friendly materials for optoelectronic devices.

## Conflict of Interests

The authors declare no conflict of interest.

1

## Supporting information

As a service to our authors and readers, this journal provides supporting information supplied by the authors. Such materials are peer reviewed and may be re‐organized for online delivery, but are not copy‐edited or typeset. Technical support issues arising from supporting information (other than missing files) should be addressed to the authors.

Supporting Information

## Data Availability

The data that support the findings of this study are available on request from the corresponding author. The data are not publicly available due to privacy or ethical restrictions.
