# Supplementary material for: Enhancing Stability of Microwave‐Synthesized Cs2SnxTi1‐xBr6 Perovskite by Cation Mixing
Source: ChemSusChem. 2024 Dec 19;18(9):e202402073. doi: 10.1002/cssc.202402073 (PMC12051252; doi:10.1002/cssc.202402073)
Supplement: Supplementary file 1 — Supporting Information [file CSSC-18-e202402073-s001.pdf]

ChemSusChem

Supporting Information

Enhancing Stability of Microwave-Synthesized $Cs_2Sn_xTi_{1-x}Br_6$ Perovskite by Cation Mixing

Emmanuel Reyes-Francis, Beatriz Julián-López,* Carlos Echeverría-Arrondo, Jhonatan Rodríguez-Pereira, Diego Esparza, Tzarara López-Luke, Jaime Espino-Valencia, Daniel Prochowicz, Iván Mora-Seró,* and Silver-Hamill Turren-Cruz*

Supplementary information

Enhancing Stability of Microwave-Synthesized $Cs_2Sn_xTi_{1-x}Br_6$ Perovskite by Cation Mixing

Emmanuel Reyes-Francis[1,2], Beatriz Julián-López[2], Carlos Arredondo Echeverría[2], Jhonatan Rodríguez-Pereira[3,4], Diego Esparza[5], Tzarara López-Luke[1], Jaime Espino-Valencia[6], Daniel Prochowicz[7], Iván Mora-Seró[2]* and Silver-Hamill Turren-Cruz[7]**

[1] *Instituto de Investigación en Metalurgia y Materiales, Universidad Michoacana de San Nicolás de Hidalgo, Edificio U, Ciudad Universitaria, Morelia, Mich, C.P. 58030, México.*

[2] *Institute of Advanced Materials (INAM), Universitat Jaume I, Av. Sos Baynat, s/n, 12071, Castelló de la Plana, España.*

[3] *Center of Materials and Nanotechnologies, Faculty of Chemical Technology, University of Pardubice, Nam. Cs. Legii 565, 53002 Pardubice, Czech Republic.*

[4] *Central European Institute of Technology, Brno University of Technology, Purkyňova 123, 612 00 Brno, Czech Republic.*

[5] *Unidad Académica de Ingeniería Eléctrica, Universidad Autónoma de Zacatecas. Jardín Juárez 147, Zacatecas Centro, C.P. 98000, Zacatecas, Zacatecas, México.*

[6] *Facultad de Ingeniería Química, División de Estudios de Posgrado, Universidad Michoacana de San Nicolás de Hidalgo, Edificio Q, Edificio U, Ciudad Universitaria, Morelia, Mich. C.P. 58030, México.*

[7] *Institute of Physical Chemistry, Polish Academy of Sciences, Kasprzaka 44/52, 01-224 Warsaw, Poland*

*Corresponding author: julian@qio.uji.es, sero@uji.es, shturren@ichf.edu.pl

Experimental Section

Instrumental Techniques

Microwave: The microwave synthesis occurred on an Anton Paar Monowave 400 mono-mode microwave reactor. The reactor consists of a borosilicate glass vial with a 6 to 20 mL filling capacity.

XRD: X-ray diffraction (XRD) patterns were obtained with a D8 Advance diffractometer from Bruker-AXS, with a Bragg-Brentano Θ/2Θ geometry and Cu K radiation. The data was collected from 10° to 60° with a step scan of 0.05° and a counting duration of 1.5 s/step.

SEM: The morphology of the samples was examined using a JSM-7001F Schottky Emission Scanning Electron Microscope equipped with an energy dispersive spectroscopy system INCA 250 (Oxford) and an acceleration voltage of 20 kV. The powders were directly deposited on a carbon film and coated with Au-Pt.

FTIR: The Fourier transform infrared spectra (FTIR) were obtained using a Jasco FT/IR-6200 spectrometer. The solid samples were measured using an ATR Pron One device with a diamond crystal over a spectral range of 4000 to 400 cm^{-1} and a resolution of 0.25 cm^{-1}. The FTIR spectrum pellets were created using about 0.5 mg of Cs_2TiBr_6, Sn_x powders, and 750 mg of dried KBr. These were mixed thoroughly in a mortar and pestle to ensure enough to cover the base of the pellet matrix. The mixture was then placed into a pressing machine at 5000 psi. After that, the pressed sample was carefully removed from the matrix to preserve its delicate structure. Finally, the pellets were placed in an open-air nitrogen atmosphere to maintain the integrity of the samples.

TGA: Thermogravimetric analysis (TGA) was performed on a NETZSCH TG 209F1 LIBRA instrument using a ramping rate of 5°C/min from 30°C to 600 °C, under nitrogen flow and oxygen flow.

UV-VIS: The UV-VIS absorption spectra of the powders were registered on a Cary 500 Scan UV-VIS-NIR spectrophotometer (Varian) equipped with an integrating sphere, and using $BaSO_4$ as a blank reference.

XPS. X-ray Photoelectron Spectroscopy (XPS) measurements were performed using an Omicron XM 1000 Al Kα monochromate X-ray source (1486.6 eV, FWHM = 0.26 eV) and an Omicron EA 125 energy analyser with a pass energy of 50 eV, at a photoemission angle θ of 35°. An electron neutralizer beam is used to minimize binding energy shifts. During measurements, the pressure was < 1 × 10−10 mbar. The samples were fixed with a copper double-sided conductive adhesive tape and analyzed as loaded. The spectra' peak positions and width were fitted using a Gaussian–Lorentzian function (GL). A Shirley background was employed using the CasaXPS Software. Adventitious carbon was set at 485.

Computational Methods.

XRD Simulations: The atomic structures of Cs_2TiBr_6 and its doped variants, $Cs_2Ti_{0.75}Sn_{0.25}Br_6$, $Cs_2Ti_{0.5}Sn_{0.5}Br_6$, and $Cs_2Ti_{0.25}Sn_{0.75}Br_6$, were geometrically optimized using density functional theory (DFT) simulations conducted with the Vienna Ab initio Simulation Package (VASP). These calculations were performed at the PBEsol level of theory on a 3 × 3 × 3 Monkhorst-Pack k-point grid centered at the gamma point (Γ). We utilized pseudopotentials and accounted for spin polarization in the valence electrons, with a kinetic cutoff energy of 500 eV for the plane-wave basis set.

Two convergence criteria were applied: 0.02 eV/Å for the forces acting on the individual nuclei during geometric relaxation, and 10^{-6} eV for the electronic steps. After optimizing the atomic structures, we generated in silico X-ray spectra using the VESTA program, as presented in the manuscript. Employing VESTA for X-ray diffraction (XRD) simulation is a common and valid method for comparing computational results with experimental diffraction data.

Chemicals.

The Cs_2TiBr_6 pristine hybrid $Cs_2Sn_xTi_{1-x}Br_6$ perovskite powder was synthesized via the microwave (MW) method. The chemical agents employed in this work consist of cesium bromide (CsBr, 99.99% purity, Aldrich), titanium bromide ($TiBr_4$, 99.99% purity, Aldrich), tin bromide ($SnBr_2$, 99.99% purity, Aldrich), acetonitrile (ACN, 99.8% purity, Aldrich), and toluene (TOL, 99.8% purity, Aldrich). All the acquired chemicals were used in their original form.

Synthesis of Cs_2TiBr_6 and mixed $Cs_2Sn_xTi_{1-x}Br_6$ powders:

Table 1 displays the amount in millimoles (mmol) of reagents employed in the synthesis of Cs_2TiBr_6 and $Cs_2Sn_xTi_{1-x}Br_6$ **(**x: 0, 0.25, 0.5 and 0.75) double perovskite powders.

Table 1. Amounts of reagents (in mmol) used for preparing Cs_2TiBr_6 and $Cs_2Sn_xTi_{1-x}Br_6$ powders.

Sample	CsBr (mmol)	$SnBr_4$ (mmol)	$TiBr_4$ (mmol)
Cs_2TiBr_6	2.00	0.00	3.50
$Cs_2Sn_{0.25}Ti_{0.75}Br_6$	2.00	0.90	3.50
$Cs_2Sn_{0.50}Ti_{0.50}Br_6$	2.00	1.80	3.50
$Cs_2Sn_{0.75}Ti_{0.25}Br_6$	2.00	2.70	3.50

Cs_2TiBr_6 pristine pre-synthesis:

Pristine reactive measurements and 13 ml of ACN were introduced into a glass vial (g30). This process was carried out within a glove box, under a vacuum with a pressure of 4.0 mbar, and an atmospheric nitrogen environment containing around 5.5 ppm of oxygen (O_2) and less than 0.1 ppm of water vapor (H_2O). Subsequently, the sealed g30 vial was continuously stirred at 5000 revolutions per minute for 70 minutes outside.

Mix cations hybrid $Cs_2Sn_xTi_{1-x}Br_6$ powders pre-synthesis:

The samples with different amounts of Sn were subjected to the same technique as the pristine pre-synthesis. Nevertheless, after a 70-minutes, the g30 vials were reintroduced to the glovebox with a controlled atmosphere environment (5.5 ppm, O_2, <0.1 ppm H_2O). The appropriate quantity of $SnBr_4$ was incorporated into the vial using MW synthesis, as indicated in Table 1. Adjusting the methodology in previous pristine experiments was necessary. It was observed that when all the chemicals were introduced simultaneously into the vial, $SnBr_2$ exhibited a prominent propensity to react with CsBr during the pre-synthesis phase, to the detriment of $TiBr_4$.

Synthesis of Cs_2TiBr_6 pristine and hybrid mix cations $Cs_2Sn_xTi_{1-x}Br_6$ powders by MW method.

Subsequently, the sealed glass g30 vial was transported to the MW reactor and heated to a temperature of 210°C for 30 minutes. The suspension was subjected to two rounds of centrifugation. In the initial washing step, an equal volume of TOL, relative to ACN, was introduced in a volume 1:1 ratio. The mixture was then subjected to centrifugation at 12,000 rpm for 10 minutes. The residual quantity was removed during a subsequent washing step, followed by adding 5 ml of TOL to the precipitate. The mixture was then centrifuged at 10,000 rpm for 5 minutes. Therefore, the precipitate that had been dissolved was subjected to quenching in an ice bath for 20 minutes. Afterward, the resulting precipitate was carefully transported to the glovebox and subjected to a heating process at 130°C for 18 hours, forming Cs_2TiBr_6 powder.

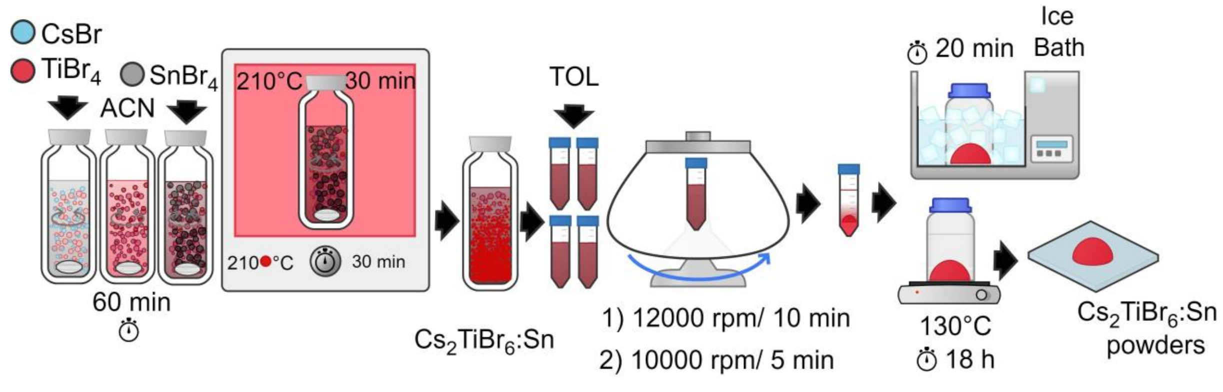

Figure S1. Schematic diagram of the MW-mediated synthesis of $Cs_2Ti_{1-x}Sn_xBr_6$ powders

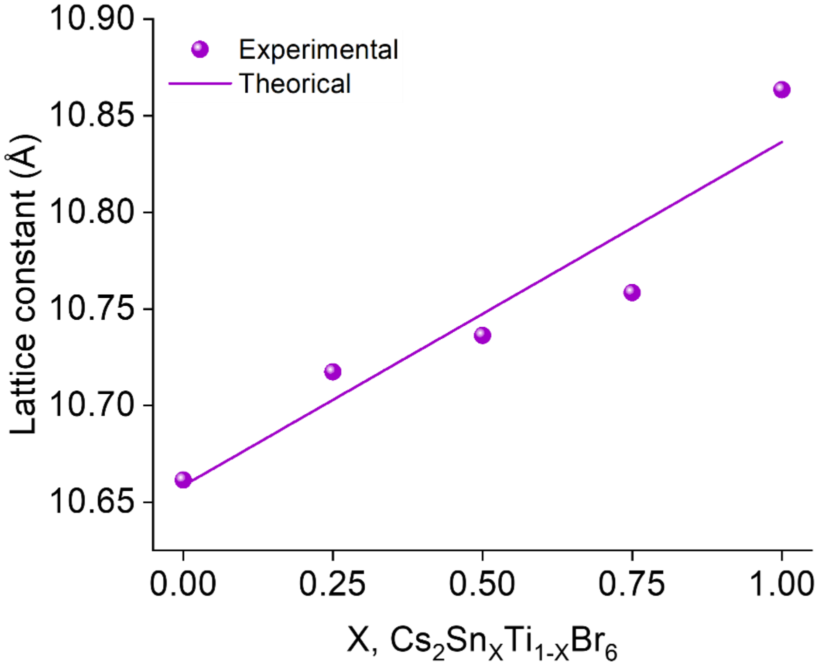

Figure S2. Lattice constant of mixed cation Sn/Ti double perovskite $Cs_2Sn_xTi_{1-x}Br_6$ using Vegard's Law

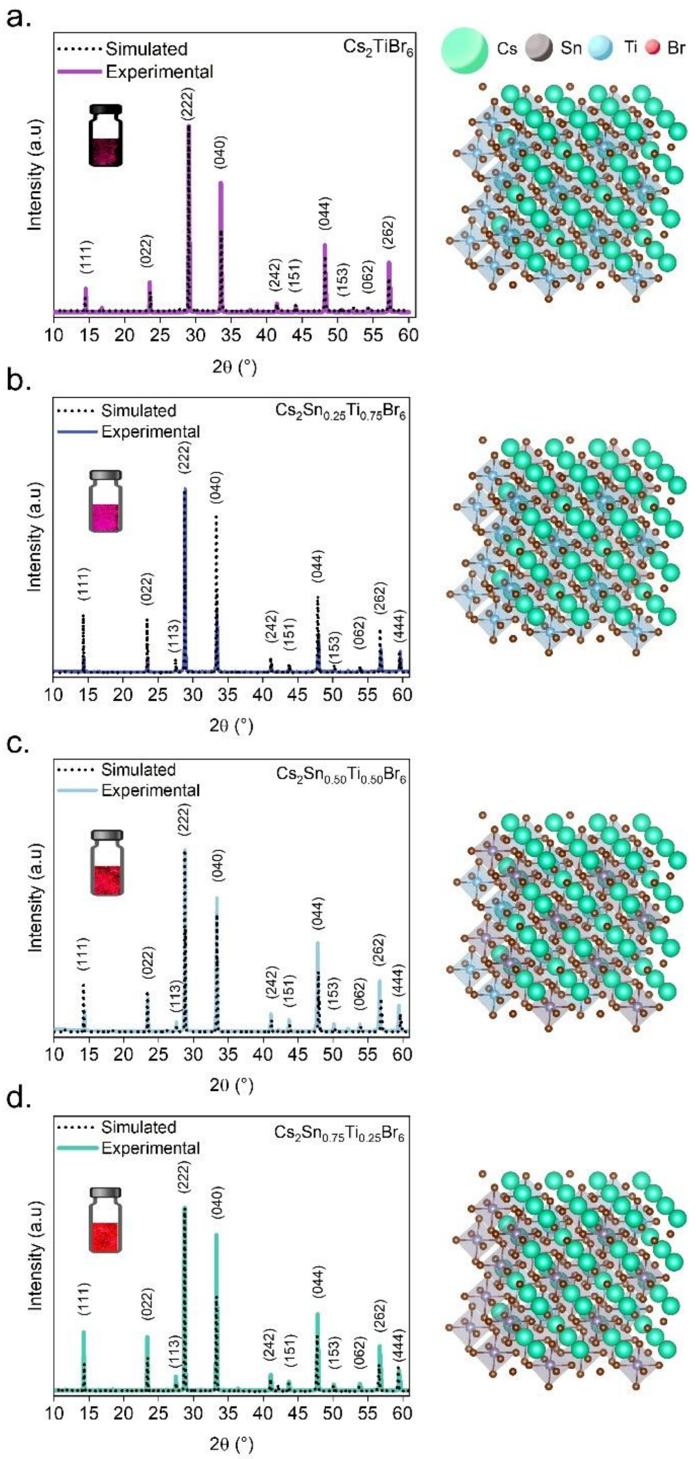

Figure S3. XRD experimental/simulated of a) Cs_2TiBr_6, b) $Cs_2Ti_{0.75}Sn_{0.25}Br_6$, c) $Cs_2Ti_{0.50}Sn_{0.50}Br_6$, and d) $Cs_2Ti_{0.25}Sn_{0.75}Br_6$, containing a colored picture of the powders contained in vials. On the right side, the corresponding crystalline structure of each diffractogram is included.

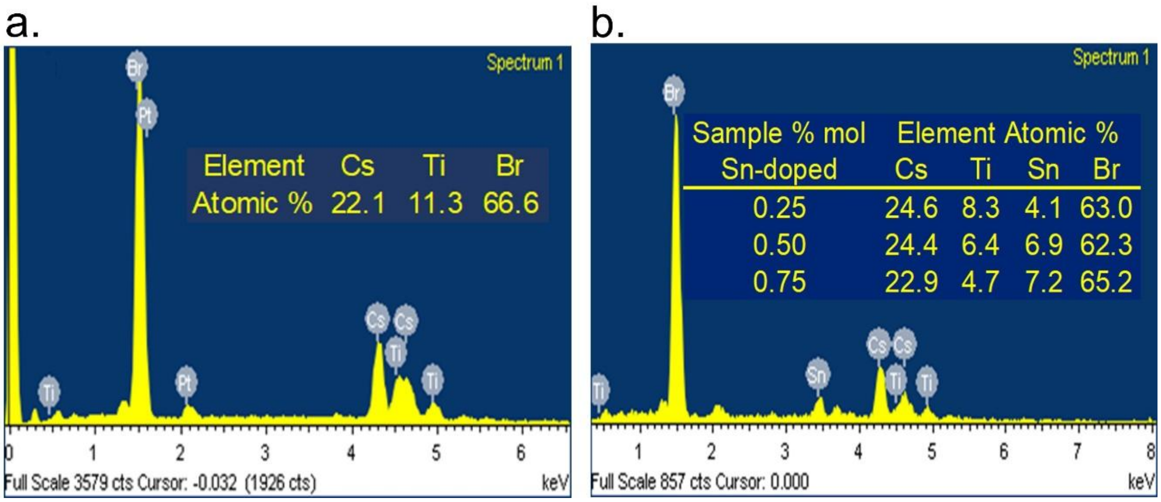

Figure S4. EDS of the a) Cs_2TiBr_6 and b) $Cs_2Ti_{1-x}Sn_xBr_6$ powders, where the inset shows Cs, Sn, Ti, and Br element proportion (expressed in atomic percentage).

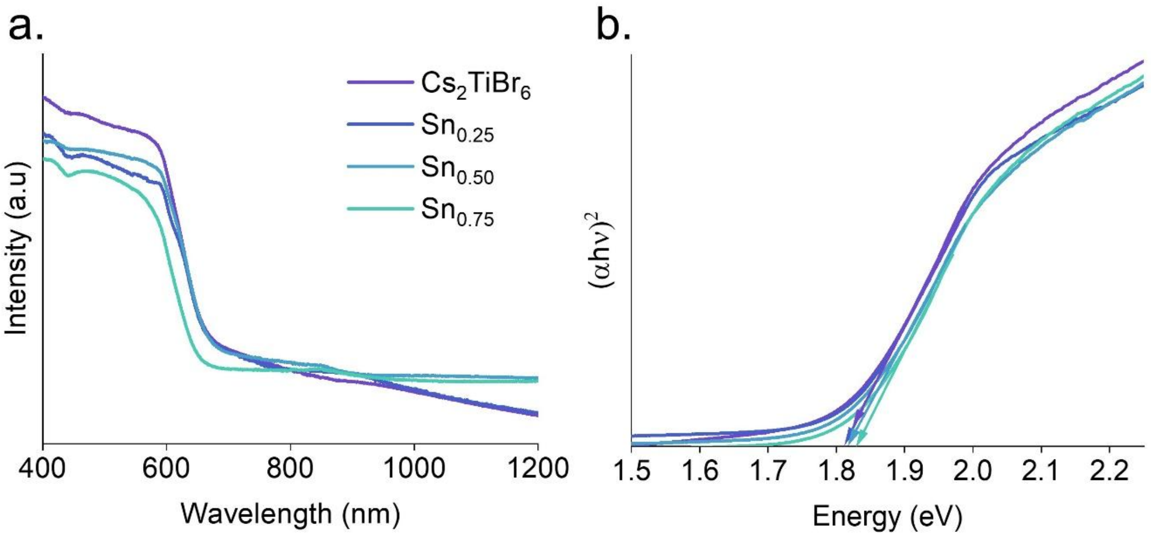

Figure S5. a) Absorption spectra and b) Tauc Plot of the Pristine and Sn-doped Cs_2TiBr_6 powders.

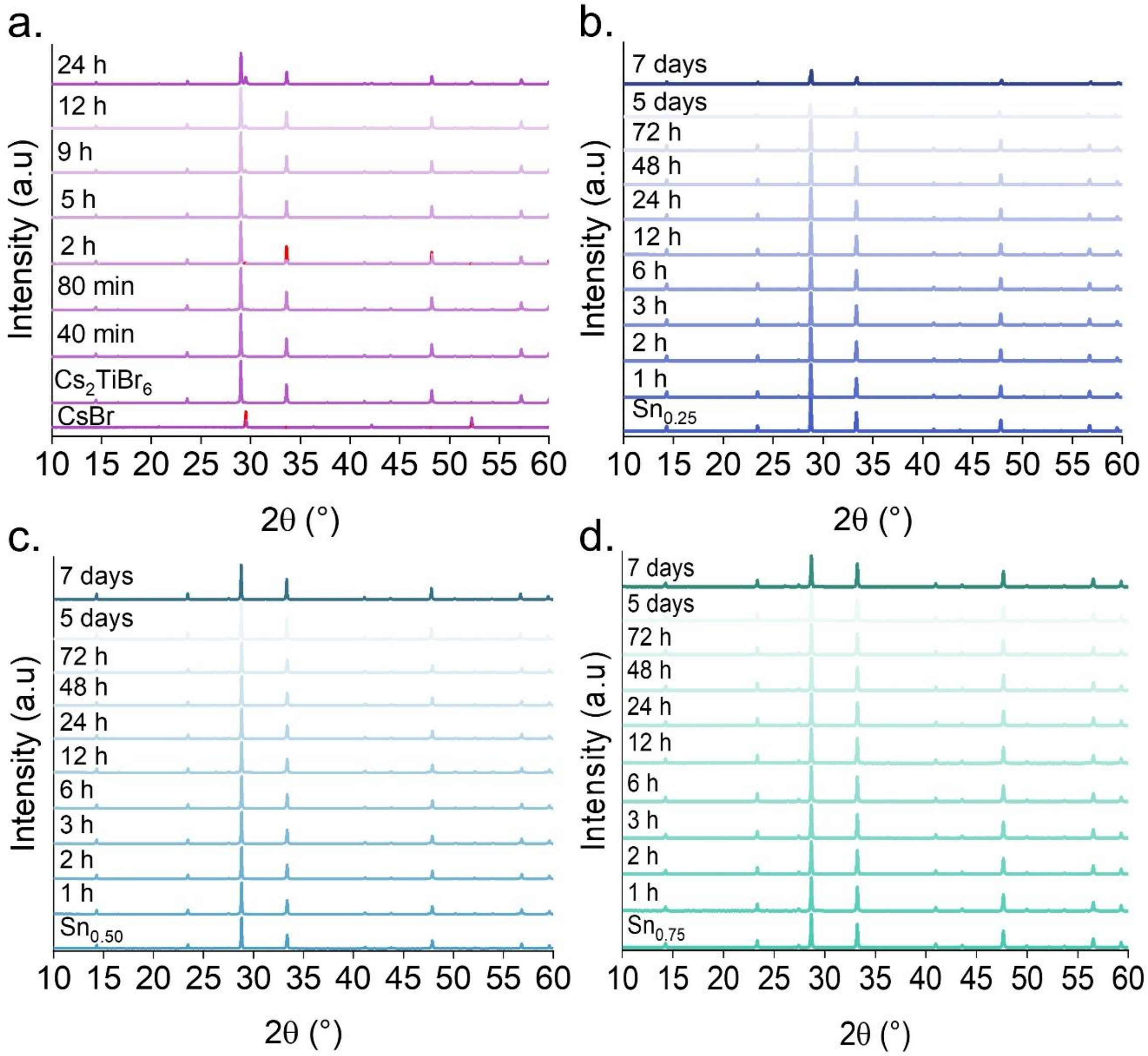

Figure S6. XRD analysis for $Cs_2Sn_xTi_{1-x}Br_6$ over time in an ambient atmosphere (20-23°C, 30-35% RH). a) Pristine, b) x=0.25, c) x=0.50 and d) x= 0.75

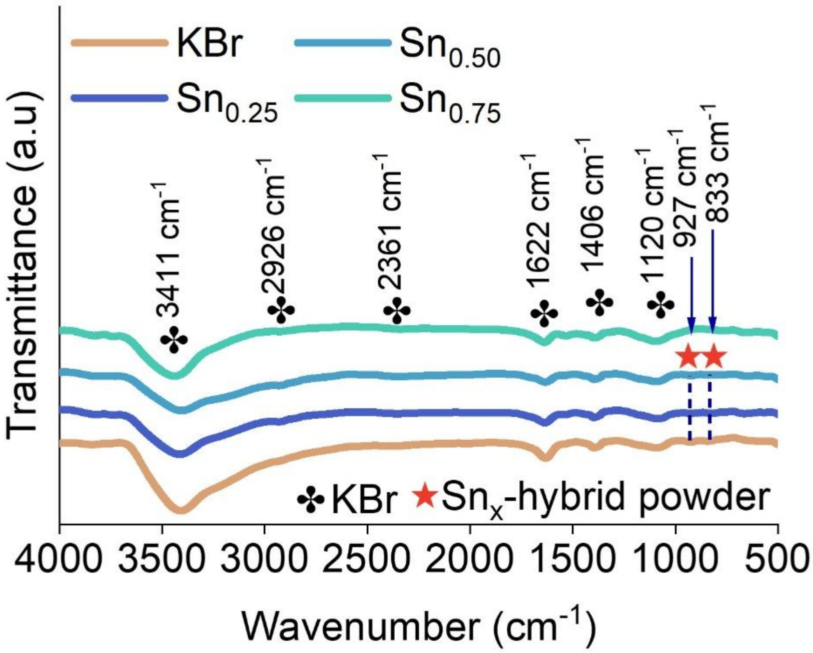

Figure S7. FTIR spectra of the pristine $Cs_2Ti_{1-x}Sn_xBr_6$ powders in the initial state of the powder pellet.
